# Clinical outcomes of closed, displaced phalangeal neck fractures in children with different types of kirschner wire fixation: A retrospective observational study

**DOI:** 10.3389/fped.2023.1039415

**Published:** 2023-02-27

**Authors:** Chunxing Wu, Jun Song, Bo Ning, Yueqiang Mo, Dahui Wang

**Affiliations:** Department of Pediatric Orthopaedics, Children's Hospital of Fudan University, & National Children's Medical Center, Shanghai, China

**Keywords:** children, phalangeal neck, fracture, longitudinal and slanting, kirschner wire

## Abstract

**Objectives:**

Inappropriate treatment of Closed displaced phalangeal neck fractures (CDPNF) in children usually leads to poor outcomes.This study was to evaluate the clinical outcomes of closed/open reduction, age, and different types of fracture and Kirschner wire (K-wire) fixation in the treatment of CDPNF.

**Materials and methods:**

Participants: Sixty patients (male, 46 and female,14; right-handed, 35 and left-handed, 25; mean age, 7.9-years-old [range, 1.0–14.5 years]) who had CDPNF were included. Preoperative x-rays showed that the fractures were displaced and exhibited obvious deformities. **Interventions**: First, reduction (four cases of open reduction and 56 cases of closed reduction) was performed followed by percutaneous K-wire fixation (cross fixation, 24 cases; longitudinal and slanting fixation, 17 cases; homolateral fixation, four cases; and single longitudinal fixation, 15 cases,) and immobilized by cast. x-ray examination following removal of the K-wires showed that the fractures were healed; the criteria for fracture healing were callus formation and the absence of fracture lines. Clinical outcome and radiographs between groups were compared.

**Results:**

According to the visual analogue scale, the pain scores were excellent. According to the Al-Qattan Grade system(AGS),all the patients presented with closed, type II phalangeal neck fractures,the results were excellent in 36 cases (36/60, 60%), good in 15 cases (15/60, 25%), fair in 5 cases (5/60, 8.3%) and poor in 4 cases (4/60, 6.7%). There were significant differences in different fracture type groups (*P *= 0.013*), operation age groups (*P *= 0.025*) and open/closed reduction groups (*P *= 0.042*). There was no significant difference in K-wire fixation type groups (*P *> 0.05).

**Conclusions:**

Patients with open reduction, the more serious fracture type, the older at the operation, were more likely to have poor AGS result. Different K-wire fixation types for CDPNF in children had the same satisfactory results.

## Introduction

Fractures of the phalanges are common in hand injuries in children, accounting for 2.7% of all fractures in children and 60% of hand fractures (1–4). Phalangeal neck fractures account for 13% of finger fractures in children (5). The phalangeal head is involved in the formation of interphalangeal joints. Improper treatment of phalangeal neck fractures often result in the appearance of deformity and limited function, which seriously affects a child's daily life and work in the future (6).

Because of the unique anatomy characteristics of the phalangeal neck in children and poor postoperative compliance, the treatment of phalangeal neck fractures in children is not the same as that in adults, and can even be more complex than that performed in adults.

This study reviewed and analyzed closed displaced phalangeal neck fractures (CDPNF) in our hospital over the past 5 years, and discussed the influence of different treatment plans, internal fixation types, injury age, fracture position, and other factors on the treatment effect.

## Materials and methods

### Patients

This study was a retrospective review and was approved by our hospital's institutional review board (IRB No. 2021–121). From December 2013 to May 2018, sixty children (60 fingers) with CDPNF were recruited. Patients with associated open wounds, pathological fracture, age more than 18y were excluded from the study.

The preoperative appearance was obviously skewed and rotated, and x-ray examination (antero-posterior radiographic and lateral view) confirmed that it was displaced. Open and closed reductions were performed by senior Pediatric Orthopedists on four fingers and 56 fingers, respectively.

### Treatments

The reduction methods were divided into open and closed reduction. First, Closed reduction should be done in the operating room by a senior pediatric orthopedist with the help of C-arm fluoroscopy and general anesthesia, every attempt should be made to avoid open reduction. If closed reduction is not successful, open reduction is done.

**Figure 1:**
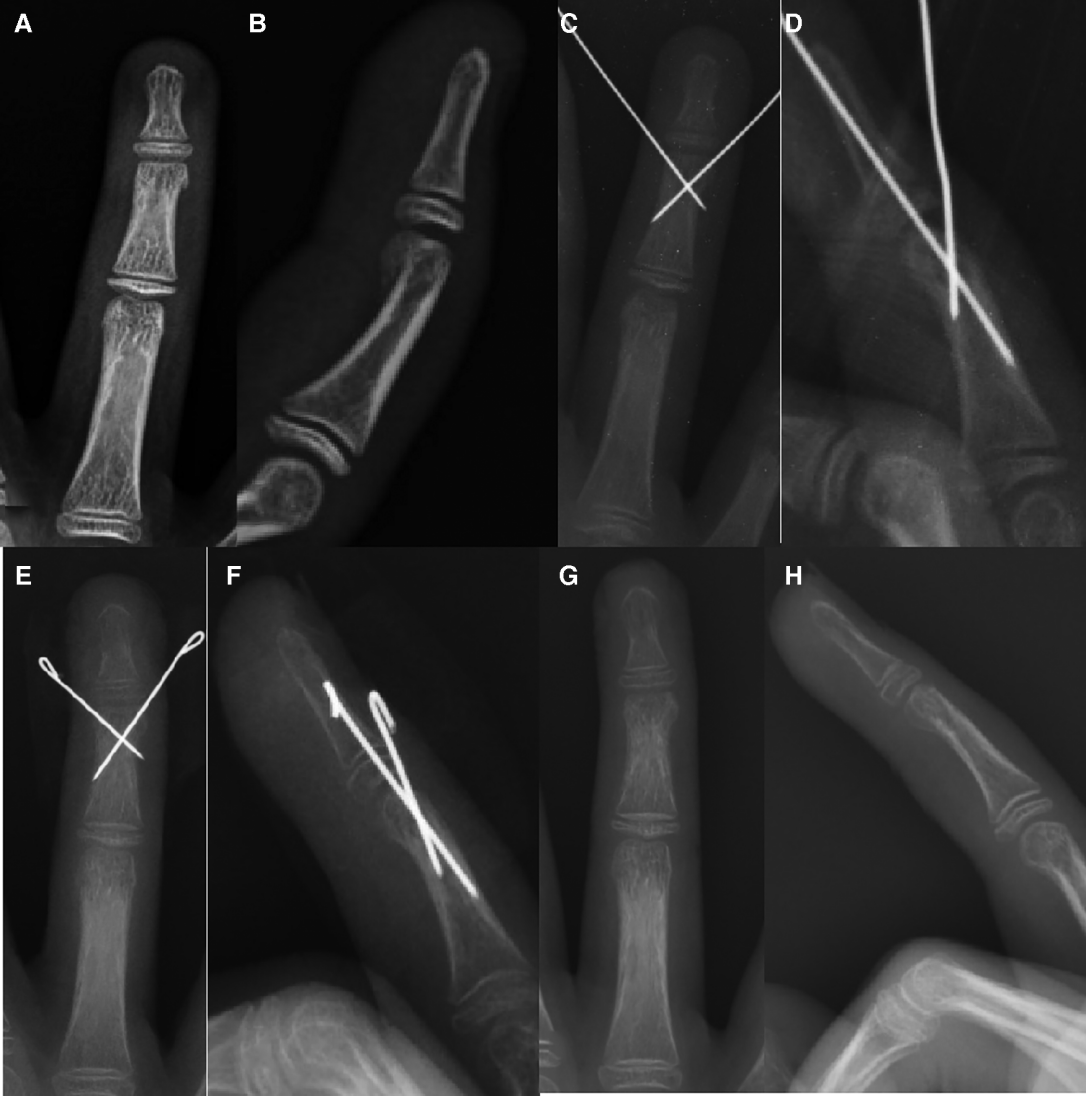
Cross fixation: 10Y11M girl with middle phalanx neck fracture of the right ring finger. (**A, B**) Eight days before operation, (**C, D**) during operation, (**E, F**) 52 days after operation, removal of K-wire, and (**G, H**) two weeks after removing the K-wire.

**Figure 2:**
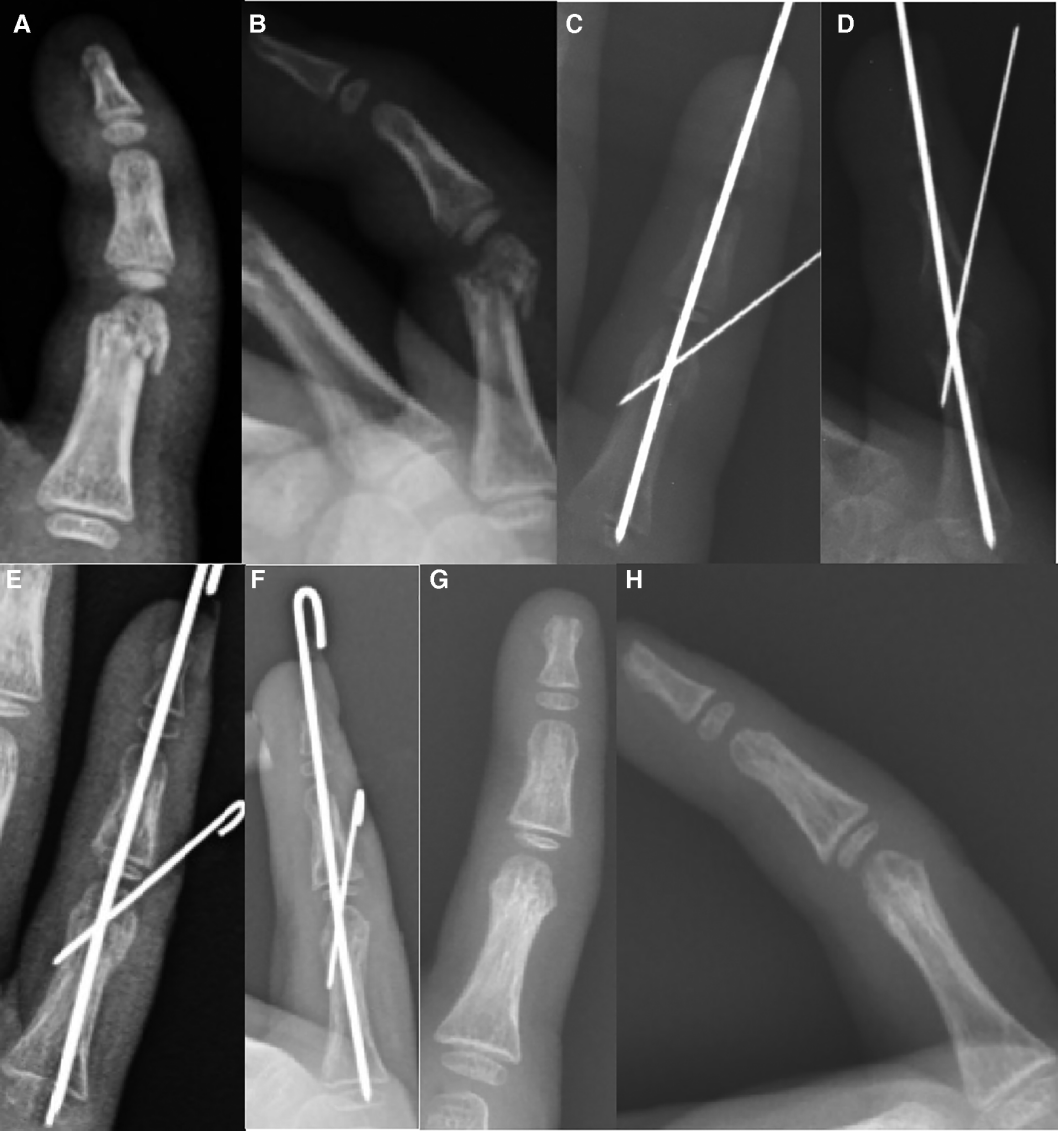
Longitudinal and slanting fixation: 7Y7M boy with proximal phalanx neck fracture of the right little finger. (**A, B**) Three days before operation, (**C, D**) during operation, (**E, F**) 52 days after operation, removal of K-wire, and (**G, H**) two months after removing the K-wire.

**Figure 3:**
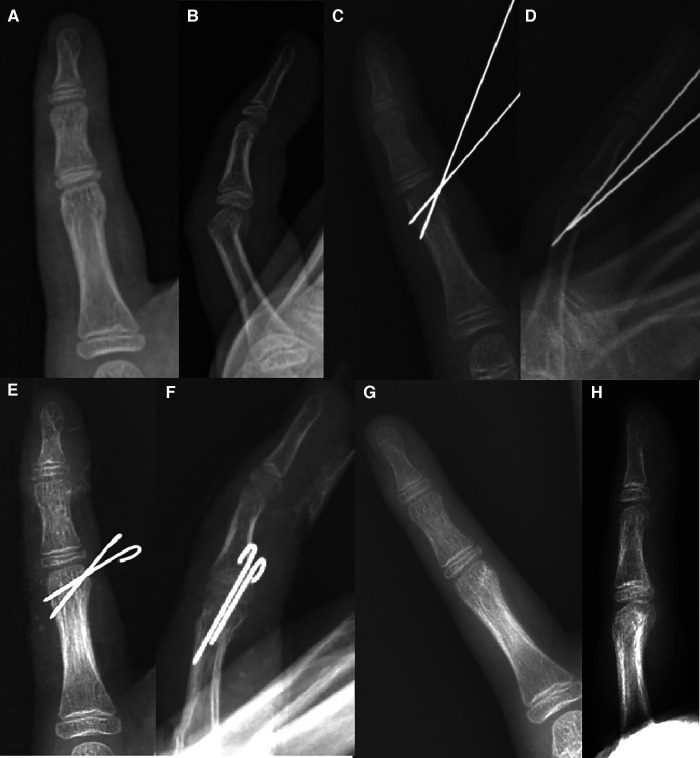
Homolateral fixation: 9Y10M boy with proximal phalanx neck fracture of the left little finger. (**A, B**) Eight days before operation, (**C, D**) during operation, (**E, F**) 51 days after operation, removal of K-wire, and (**G, H**) three months after removing the K-wire.

**Figure 4:**
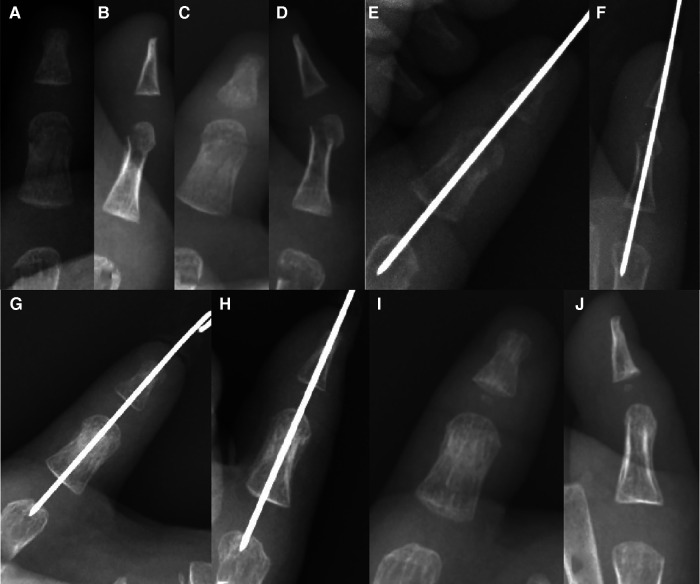
Single longitudinal fixation: 1Y6M boy with proximal phalanx neck fracture of the left thumb. (**A, B**) Eight days before operation, (**C, D**) two days before operation, (**E, F**) during operation, (**G, H**) 41 days after operation, removal of K-wire, and (**I, J**) two weeks after removing the K-wire.

After reduction, Kirschner wires (K-wires) with diameters of 0.6, 0.8, and 1.0 mm were selected for internal fixation according to the thickness of the phalanges. Lateral and longitudinal approaches were used for the K-wires. The lateral approach is close to the fracture line, with the intersection of the dorsum and the lateral side with the finger as the entry point. After entering the distal bone cortex of the fracture vertically through the skin, the direction is adjusted to the oblique approach, and the fracture line is passed to attain the opposite proximal bone cortex of the fracture. The longitudinal approach is from the distal end of the finger to the proximal phalanx base, passing through the distal and middle phalanx. Depending on the classification of fractures, there are four different wire fixation methods using lateral and longitudinal approaches: cross fixation (CF) (Figure 1), longitudinal and slanting fixation (LSF) (Figure 2), homolateral fixation (HF) (Figure 3), and single longitudinal fixation (SLF) (Figure 4).

### Assessment

After phalangeal neck fracture was reduced and fixed with K-wire, the K-wire was cut short, the exposed K-wire end was sealed with a sterile dressing and phalanx was immobilized with plaster (7). The patients visited the hospital at first 2, 4, 6, 8, 10 week (every 2 weeks) and later every 1 month (after 10 weeks) for assessment of the stability and a radiograph (anterior-posterior and lateral view) was obtained.

The criteria for fracture healing were callus formation and fracture line blurring seen by x-ray, the K-wire was not removed until the fracture has healed. After removing the K-wire, the children were encouraged to practice finger flexion and extension independently to facilitate recovery of the fingers. One month after K-wire removed, if the finger was stiff, had limitations in mobility, the patient will undergone physiotherapy treatment. The appearance and function of the affected fingers were evaluated later. Pain was assessed by the visual analog scale(VAS) (8). Radiographic and clinical outcomes were retrospectively graded by the Al-Qattan grading system, including union, deformity, range of motion, and function(AGS) (Table 1) (9).

**Table 1:** Classification of final results following phalangeal neck fractures in children (Al-Qattan's grading system).

Classification		Excellent	Good	Fair	Poor
	**Union**	Yes	Yes	Yes	Nonunion or avascular necrosis
	**Joint range of motion**	Full	>50°at IP/DIP, >90°at PIP	20–50°at IP/DIP, 50–90°at PIP	<20° at IP/DIP, < 50°at PIP
	**Residual deformity**	No	No	Mild	Severe
	**Digit function**	Normal	Good	Useful	Lost

DIP, distal interphalangeal joint of fingers; IP, interphalangeal joint of thumb; PIP, proximal interphalangeal joint of fingers.

Data were analyzed using the chi-square test, one-way analysis of variance (ANOVA test), Kruskal-Wallis H test with SPSS 19.0. The level of significance was set at *P* < 0.05.

## Results

### General clinical data

**Figure 5:**
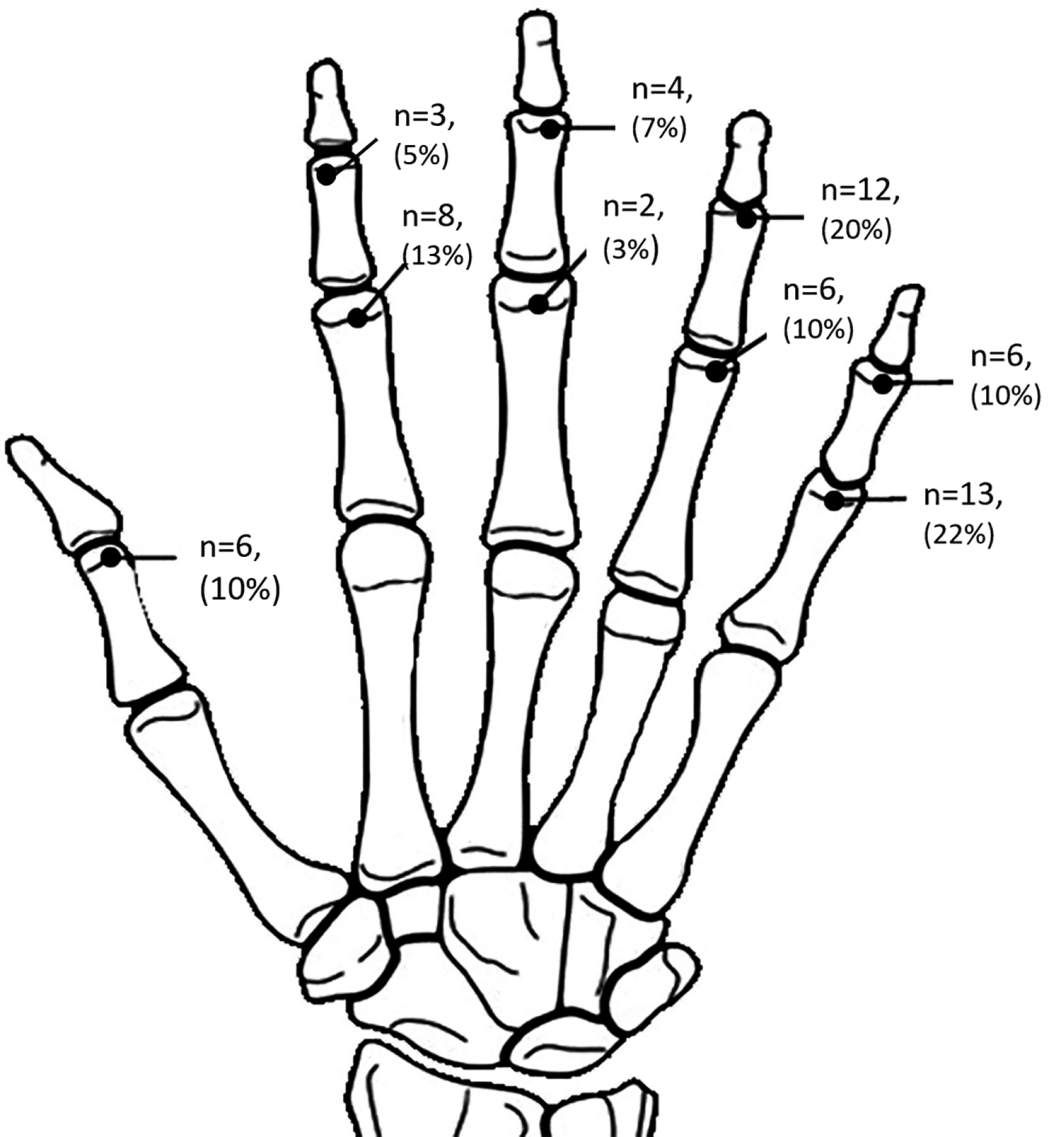
Fracture locations’ distribution.

A total of 60 cases of phalangeal neck fractures in 60 children. There was an obvious male predominance (46 boys and 14 girls, 3.3:1 ratio) and the right hand was more commonly involved than the left (35 right-handed and 25 left-handed, 1.4:1 ratio). The mechanism of injury includes entrapment of the digit in a closing door or heavy weights (20 cases, 33.3%), sports such as basketball (19 cases, 31.7%) and falling (21 cases, 35.0%). The ring fingers and little fingers were affected the most and the thumb and long finger the least. The distribution of fracture locations is shown in Figure 5.

**Figure 6:**
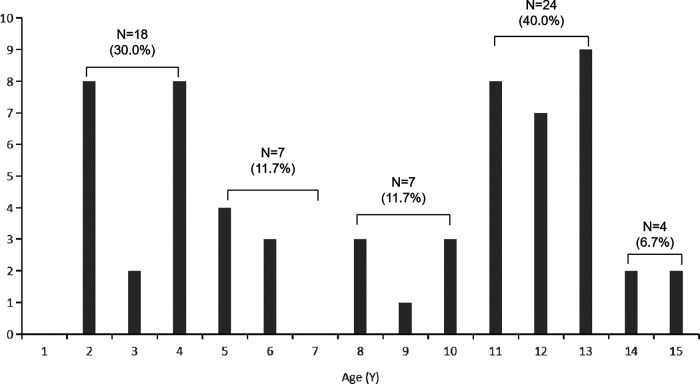
Age distribution of patients.

Young adolescents between 10 and 13 years of age comprised 40.0% (24 fingers) of the series, and young children between 1 and four years of age comprised 30.0% (18 fingers) of the series (Figure 6).

The average operation age was 7.9 years (range, 1.0–14.5 years). The average interval between injury and operation was 3.3 days (0–16 days). The average follow-up time was 24 months (range, 7–60 months), and the average postoperative K-wire removal time was 52.0 days (28–132 days).

There were no K-wire fractures, nonunion and avascular necrosis.

### Classification of fractures

The most accepted grading system in the assessment of outcome of management of phalangeal neck fractures is Al-Qattan's grading system.

According to Al-Qattan's extended classification of types of phalangeal neck fractures, type I undisplaced, type II displaced with some bone-to-bone contact, and type III displaced with no bone-to-bone contact. Type II fractures are sub-classified into four sub-types according to fracture configuration. A type IIa fracture has a transverse fracture line at the neck of the phalanx. A type IIb fracture has an oblique fracture line. Type IIc fractures have a dorsal or a dorso-lateral bony flange. A type IId fracture has a small distal fragment and is frequently seen in very young children (9–12). All the phalangeal neck fractures are classified into type II: type IIa (38 fingers), type IIb (eight fingers), and type IIc (14 fingers).

### Outcomes of the different treatments

There was no significant difference in muscle strength or sensation between injured and healthy fingers in the 60 cases; 60 fingers were all excellent according to VAS.

According to AGS, we compared the duration from injure to operation, operation time, right/left hand, affected finger (thumb, index finger, middle finger, ring finger, little finger), affected phalanx (distal, middle, proximal), fracture healing time, finger recovery time after removal of K-wire, which revealed no significant difference (multiple linear regressions, ANOVA), except fracture types (ANOVA, *P *= 0.002) and operation age.

According to AGS, thirty-six fingers were excellent, fifteen fingers were good, five fingers were fair, and four fingers were poor.

In terms of different operation age, in the “1–4 y” group, there was only one “fair” finger (5.6%,1/18) no “poor” fingers (0%,0/18); in the “4–7y” group, there was one “fair” finger (14.3%,1/7) and no “poor” fingers (0%,0/7); in the “7–10y” group, there was one “fair” finger (14.3%, 1/7) and no “poor” finger (7.1%,1/14); in the “10–13y” group, there were two “fair” fingers (8.3%, 2/24) and two “poor” fingers (8.3%,2/24); in the “over 13y” group, there were one “fair” finger (25%, 1/4) and one “poor” fingers (25%,1/4). Kruskal-Wallis Test was used to compare different fracture-type groups, there was a significant difference in AGS outcomes (*P* = 0.025*). We found the trends, the patients with the older at the operation, were more likely to have poor AGS (Table 2).

**Table 2:** Comparison of Al-Qattan's outcomes of different classification (fracture type groups, operation age groups and open/closed groups).

Classification		Excellent	Good	Fair	Poor
**Results**
**Fracture type** (Kruskal-Wallis Test) (*P *= 0.013*)	**II a** (N = 38)	27 (71.1%)	9 (23.7%)	1 (2.6%)	1 (2.6%)
	**II b** (N = 8)	5 (80.0%)	1 (12.5%)	0 (0%)	2 (25.0%)
	**II c** (*N* = 14)	4 (28.5%)	5 (35.7%)	4 (28.6%)	1 (7.1%)
**Operation age** (Kruskal-Wallis Test) (*P *= 0.025*)	** 1∼4y** (*N* = 18)	15 (83.3%)	2 (11.1%)	0 (0%)	1 (5.6%)
	**4∼7y** (*N* = 7)	1 (14.3%)	5 (71.4%)	1 (14.3%)	0 (0%)
	**7∼10y** (*N* = 7)	4 (57.1%)	2 (28.6%)	1 (14.3%)	0 (0%)
	**10∼13y** (*N* = 24)	15 (62.5%)	5 (20.8%)	2 (8.3%)	2 (8.3%)
	**13∼15y** (*N* = 4)	1 (25.0%)	1 (25.0%)	1 (25.0%)	1 (25.0%)
**Open/Closed**	**Open** (*N* = 4)	2 (50%)	0 (0%)	2 (50%)	0 (0%)
	**Closed** (*N* = 56)	34 (60.7%)	15 (26.8%)	3 (5.4%)	4 (7.1%)
		**Excdllence + Good**		**Fair + Poor**	
**Open/Closed** (Chi-square test, *P *= 0.042*)	**Open** (*N* = 4)	2 (50%)		2 (50%)	
	**Closed** (*N* = 56)	49 (87.5%)		7 (12.5%)	

In terms of different fracture types, in the “type IIa” group, there were two “fair” fingers (2.6%,1/38) and one “poor” finger (2.6%,1/38); in the “type IIb” group, there were no “fair” finger (0%,0/8) and two “poor” fingers (25%,2/8); in the “type IIc” group, there were no “fair” finger (28.6%, 4/14) and one “poor” finger (7.1%,1/14). Kruskal-Wallis Test was used to compare different fracture-type groups, there was significant difference in AGS outcomes (*P *= 0.013*). We found the trends, the patients with more serious fracture, were more likely to have poor AGS (Table 2).

According to AGS, in Open operation group, 2 patients got AGS “Excellence or poor”, 2 patients got AGS “Fair or poor “. In close operation group, 49 patients got AGS “Excellence or poor”, 7 patients got AGS “Fair or poor”. The Chi-square test (Pearson's method) was used to compare open and close group, there was significant difference in AGS outcomes (Pearson's method, *P *= 0.042*) (Table 2).

In terms of K-wire fixation types, in the “fair” group, four fingers were fixed by CF (16.7%, 4/24), finger were fixed by HF (25%, 1/4), none of the fingers were fixed by LSF (0%, 0/17) and SLF (0%, 0/15). In the “poor” group,four fingers were fixed by CF (16.7%, 4/24), finger were fixed by HF (25%, 1/4), none of the fingers were fixed by HF (0%, 0/4), LSF (0%, 0/17) and SLF (0%, 0/15). We chose the appropriate K-wire fixation type to sustain stable after fracture reduction, depending upon the fracture types. So there was no significant difference in AGS outcomes (Kruskal-Wallis H test, *P *= 0.106) in different K-wire fixation groups (Table 3). We compared the fracture healing time (ANOVA, LSD method, *P *= 0.451), and finger recovery time after removal of K-wire during four different wire fixation types, which also revealed no significant difference (ANOVA, LSD method, *P *= 0.760) (Table 3).

**Table 3:** Comparison of AGS, fracture healing time, and finger recovery time between different kinds of K-wire fixation.

Fixation type		Cross (*N* = 24)	Longitudinal and slanting (*N* = 17)	Homolateral (*N* = 4)	Single Longitudinal (*N* = 15)
**AGS Results** *(Kruskal-Wallis Test, P *= 0.106)	**Excellent**	11 (45.9%)	11 (64.7%)	3 (75.0%)	11 (73.3%)
	**Good**	5 (20.8%)	6 (35.3%)	0 (0%)	4 (26.7%)
	**Fair**	4 (16.7%)	0 (0%)	1 (25.0%)	0 (0%)
	**Poor**	4 (16.7%)	0 (0%)	0 (0%)	0 (0%)
**Fracture healing time** (day) (ANOVA, LSD method, *P *= 0.451)		47.58	50.12	62.00	56.93
**Finger recovery time after removal of K-wire** (day) (ANOVA, LSD method, *P *= 0.760)		40.00	48.41	31.00	29.60

## Discussion

Many researchers believe that phalangeal fractures in children have strong bone shaping ability and do not require anatomical reduction. In clinical practice, it is not rare for patients with inaccurate fracture fixation and fracture displacement to obtain conservative treatment (11, 13–17). The phalangeal head in children is primarily cartilaginous and has no tendon attachments. Once the fracture of the phalanges neck is displaced or rotated, it will eventually lead to malunion, often causing finger deformity and joint rigidity, and affecting function (13–15, 18). Therefore, for children whose fingers are angulated, rotated, limited in function, unable to cooperate, and have poor compliance, more doctors are recommending the use of internal fixation (11, 19).

Following phalangeal neck fractures, the phalangeal head loses all its periostal attachments and is relatively ischemic (9). The main source of blood supply is through the collateral ligaments near the phalangeal neck. Thus, certain precautions should be taken to minimize the risk of avascular necrosis of the phalangeal head including minimal handing with preservation of collateral ligament attachments during open reduction, and avoiding multiple passes of K-wires, which will cause thermal injury (9, 10). Good blood circulation in closed reduction is conducive to fracture healing and good function. In this retrospective study, 50% (2/4) of the children with open reduction had “fair or poor” finger function, which was higher than that with closed reduction (12.5%, 7/56). For the reduction of unstable phalangeal neck fractures, Closed reduction and K-wire fixation should be the first choice following reduction of these unstable phalangeal neck fractures.

Wallace et al. (20) reported similar outcome in infants and older children, and think age does not seem to affect the outcome of management of type II fractures. However, our study showed age is an important factor for the recovery of finger function. With increasing age for the operation, phalanx healing gradually decrease, the patients were more likely to have poor AGS results. For older children with phalangeal neck fractures, it is necessary to start with closed reduction, early needle withdrawal, and early rehabilitation so as to improve the possibility of functional recovery as much as possible.

After reduction, the choice of internal fixation is different. For adult phalangeal fractures, steel plates, screws, steel wires, tension bands, external fixation frames, syringe needles, and K-wires can be used. However, for phalangeal neck fractures in children, because of their small size, phalanges in children require more sophisticated internal fixation; the epiphyseal plates need be protected and not destroyed, and there are few tools available for internal fixation. The K-wire can provide relatively firm fixation, using percutaneous fixation, with little damage and high accuracy (11, 21). In addition, K-wires are available in different diameters, which can be useful given the different thicknesses of phalangeal neck fractures in children of different ages.

For phalangeal neck fractures, there are many types of K-wire fixation; e.g., CF, LSF, HF and SLF. CF is a relatively easy operation without crossing the joint, and is preferred by many doctors. However, for children, the phalangeal neck slice is small, especially in oblique fractures such as type IIb, IIc. CF sometimes can only fix one side; the other side is difficult to fix. When homolateral double wire fixation is adopted, the fracture slice is too small and there is no enough space for internal fixation. If CF or HF is successful, the K-wire is easy to withdraw, because children are often uncooperative and move their fingers.

For oblique phalangeal neck fractures that are difficult to fix, we suggest the LSF or SLF. LSF is difficult to master and requires more practice and experience but has many advantages. First, when the longitudinal K-wire reaches the distal end of the fracture, it cannot only fix the fracture through the fracture line, but the distal end of the fracture can also be reduced by adjusting the K-wire. Second, the oblique K-wire can prevent rotation at the fracture. Third, following insertion, the longitudinal K-wire is firmly fixed at the fracture site. Even if the child does not cooperate, the longitudinal K-wire is difficult to withdraw from the fracture site. If the fracture occurs in the middle phalanx neck, some pediatric orthopedist will prefer to choose SLF.

Is the longitudinal K-wire crossing multiple interphalangeal joints affect joint motion? This retrospective study showed that appearance and functional recovery following LSF and SLF were generally satisfactory. There was no significant difference in four-wire fixation types. We compared fracture healing time and finger recovery time after removal of K-wire during four different wire fixation types, which also revealed no significant difference (Table 3).

The duration of fixation of cap fractures is controversial. The K-wires are removed in three weeks in most pediatric hand fractures (10). Children do not cooperate and do not pay attention to self-protection. If the K-wire is pulled out early, re-fracture or malunion is likely to happen. Several authors (18, 22) have found that re-displacement sometimes occurs in children with phalangeal neck fractures if the fixation is removed during this time. Hence, Leonard et al. (18) recommended retaining the fixation for six weeks. Al-Qattan (10) and Matzon et al. (19) recommended for 4–5weeks. Therefore, the K-wire should not be removed through the x-ray show's callus formation and blurring of the fracture line. In our series, the K-wires were removed at 7–8 weeks and there were no re-displacements.

In conclusion, the overall recovery of the fractures of the proximal and middle phalangeal neck fractures was satisfactory. Patients with open reduction, more serious fracture type, the older at the operation, were more likely to have poor AGS results. Reduction of phalangeal neck fractures requires personalized treatment. There was no statistical difference compared with the four kinds' wire fixations (CF, LSF, HF, SLF). LSF can effectively prevent slipping and rotation, and is the suggested choice for complex oblique phalangeal neck fractures in children. One limit of our study was that the sample size was small, and further expansions of the research population and long-term follow-up work are needed.

## Data Availability

The original contributions presented in the study are included in the article/Supplementary Material, further inquiries can be directed to the corresponding author.
